# Jain point: an alternate laparoscopic non-umbilical first blind entry port to avoid vessel, viscera, adhesions and bowel (VVAB)

**DOI:** 10.1007/s13304-021-01099-z

**Published:** 2021-06-13

**Authors:** Nutan Jain, Vandana Jain, Anadeep Chandi, Sakshi Srivastava, Shalini Singh, N. Vasundhara

**Affiliations:** 1 Department of Obstetrics and Gynecology, GSVM Medical College, Affiliated By Kanpur University, Kanpur, India; 2 Vardhman Trauma and Laparoscopy Centre Pvt. Ltd, 3rd KM, Jansath Road, Muzaffarnagar, 251001 Uttar Pradesh India; 3 Department of Obstetrics and Gynecology, Kasturba Hospital, Affiliated By Delhi University, New Delhi, India; 4 Pandit Bhagwat Dayal Sharma Post Graduate Institute of Medical Sciences, Rohtak, India; 5 House No: 957, sector 25, Panchkula, 134109 Haryana India; 6 Rohilkhand Medical College and Hospital, Affiliated By Bareilly International University, Bareilly, India; 7 House no. 341, Chhoti Baradari Part 1, Garha Road, Jalandhar, 144001 Punjab India; 8 Institute of Medical Sciences, Affiliated By Banaras Hindu University, Varanasi, India; 9 GBH American Hospital, Udaipur, India; 10 7/571 Vikas Nagar, Aliganj, Lucknow, India; 11 MCH Gynaecological Oncology, Acharya Harihar PG Institute of Cancer, Affiliated By Utkal University, Cuttack, India; 12 THARA-53, Thekkemadom Road, Thrissur, 680001 Kerala India

**Keywords:** Palmer’s point, Jain point, Left lateral port, Non-umbilical entry, Laparoscopic entry port, Entry in previous abdominal surgeries

## Abstract

The Jain point entry is based on the concept of non-umbilical entry to avoid sudden catastrophic injury to major retroperitoneal vessels, viscera, adhesions and bowel which could happen before the start of procedure by blind umbilical entry. To study the safety and efficacy of a novel first non-umbilical blind entry port. Tertiary referral centre for advanced laparoscopic surgeries with active training and fellowship programs. A large retrospective study of 7802 cases done at Vardhman Infertility & Laparoscopy Centre from January 2011 to December 2020. In all cases, first blind entry was by veress needle and 5 mm trocar and telescope through a non-umbilical port, The Jain point, irrespective of BMI, large masses, lax abdomen, previous surgery and complex situations. Patients’ demographic profile, types of surgeries performed and entry-related complications were recorded and analysed. Mean age of patients was 33 years with BMI ranging from 12.66 to 54.41 kg/m^2^. Thus, Jain point can be applicable for all ranges of BMI, all types of surgeries from simple to complex and large masses. Entry related minor complications were in 3.4% cases while major complication involving bowel occurred in one case. No case of injury to major retro-peritoneal vessel was seen. Jain point entry is a novel, first blind 5 mm non-umbilical, entry technique in a variety of surgeries and previous scars and patients with wide range of BMI. It has a short learning curve and continues as main ergonomic working port.

## Introduction

The aim of this study is to introduce the concept of non-umbilical entry [1] to avoid catastrophic complications which could be associated with first blind umbilical entry [2]. Since last 70 years, the umbilicus is used as the preferred site for creating pneumoperitoneum and entering into abdominal cavity. However, the close proximity to the great vessels [3] in midline and possibility of unanticipated paraumbilical adhesion [4, 5] are the main drawbacks for blind umbilical entry. A large multicentric prospective study revealed [6] that the intestinal injuries and major complications during laparoscopy occur in 5.7/1000 procedures. Approximately 70% of these are related to the primary port entry. The overall incidence of laparoscopic entry injuries is 3.3/1000 with gastrointestinal damage occurring in 1.3/1000 and major vessel injuries in 1.05/1000. At least 50% of the major complications occur prior to commencement of the intended surgery meaning that they are related to first blind primary umbilical port. Hence Royal College of Obstetricians and Gynaecologists (RCOG) also opinioned that umbilicus may not be the safest point for first blind entry [7]. The non-umbilical entry has been strongly advocated by a recent article entitled ‘overview of gynaecological laparoscopic surgery and non-umbilical entry site’ on “UpToDate” [1]. It recommends non-umbilical entry in previous surgery, large pelvic masses, gross obesity or underweight patients, pregnancy, very lax abdomen, and umbilical hernia. Looking at these recent guidelines, we fall in safe zone using the non-umbilical entry technique universally in all patients.

We have used a non-umbilical primary entry site, “Jain point”, to avoid the retroperitoneal vessels, viscera, adhesion and bowel (VVAB) at the umbilicus, as a universal method in all routine cases and in abovementioned challenging situations.

## Method

This is a large retrospective study of 7802 cases conducted from January 2011 to December 2020. The study period of almost a decade gave us the opportunity to improve and modify our technique in routine and increasingly challenging situations, and at the same time making the technique easy for beginners. The study was carried out at Vardhman Infertility & Endoscopy Centre, India; which is a tertiary care centre for laparoscopic surgeries with an active fellowship program and many short-term trainees. Most of the laparoscopic entries are made by fellows and trainees, initially, under supervision of senior consultants. There is a short learning curve of 8–10 cases, after that they are making entries on their own. Hence this technique is particularly feasible for beginners and novice endoscopist who have the fear of major retroperitoneal vessel injury and bowel and visceral injuries in the initial part of their careers.

We adopted the concept of universally using non -umbilical 5 mm first trocar entry in simplest to most complex cases in all body types, thin and obese, and all variety of pelvic pathology. Clinical situations with or without a previous surgery were entered by the newer 5 mm non-umbilical port the Jain point to assess the safety, feasibility, and to establish a new technique. All the laparoscopic surgeries where “Jain point” was used for first blind laparoscopic entry and then continued as main working port were included in the study.

### Surgical technique

**Fig. 1:**
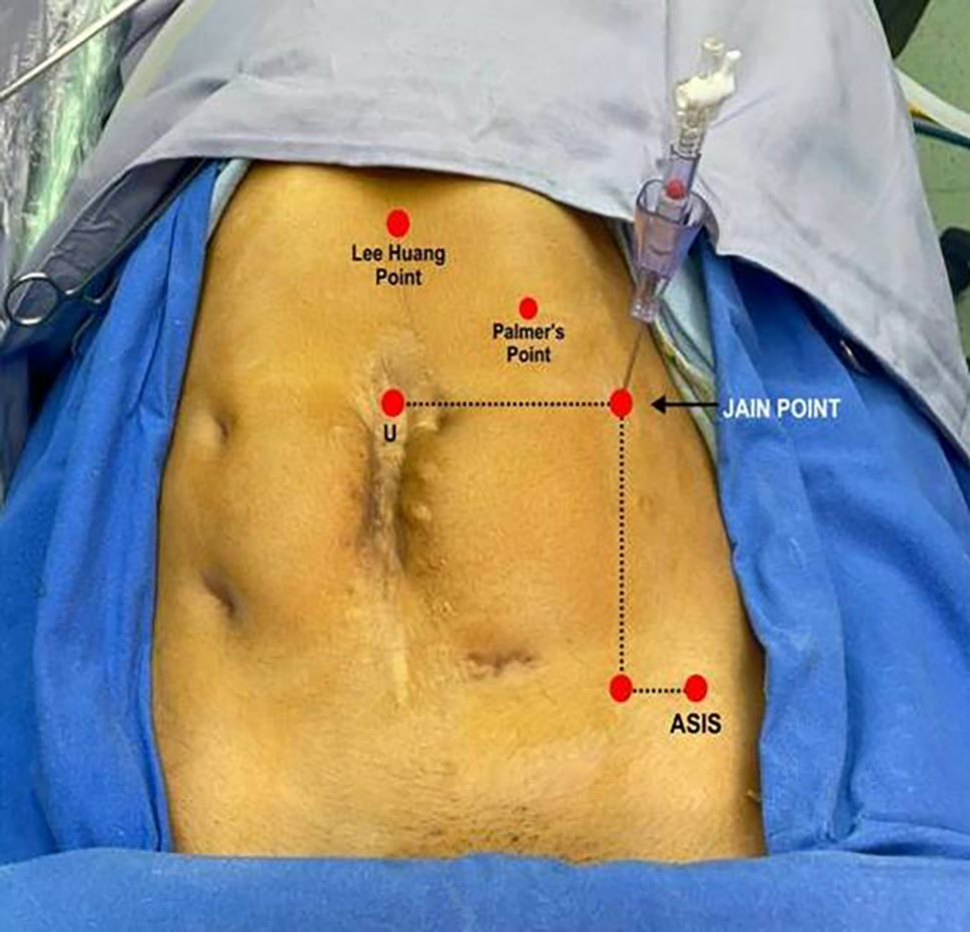
Showing relative positions of all entry ports. Jain point is lowest and most lateral being 10 to 13 cm lateral to umbilicus at L 4 level. It has a single very prominent bony landmark the ASIS. Palmers point is higher and more medial hence cannot be used as a working port

**Fig. 2:**
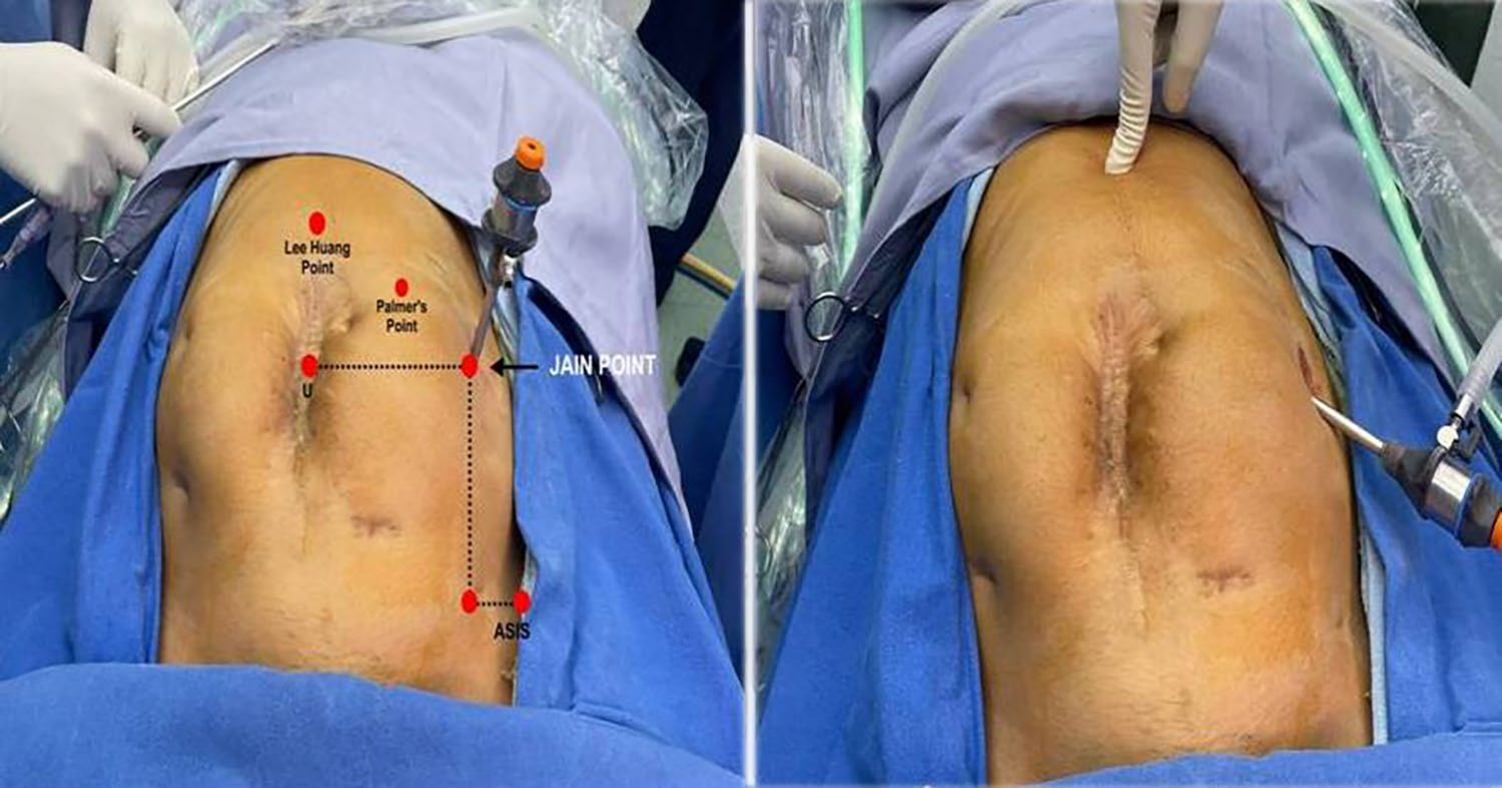
Optimizing the 10 mm port under the vision of 5mm port inserted at the Jain point, to avoid dense periumblical adhesions in this case

**Fig. 3:**
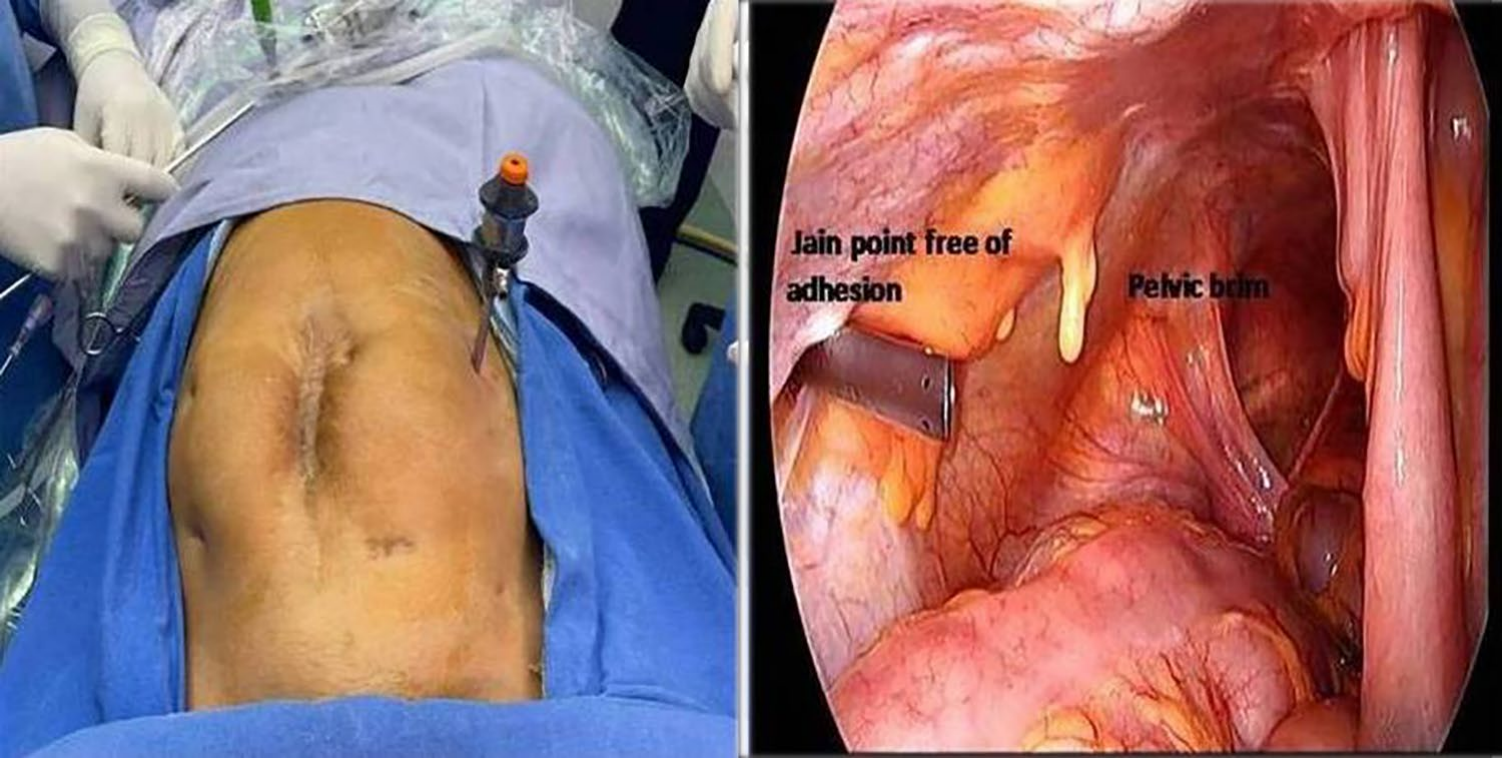
Showing Jain Point port coming from adhesion free area

**Fig. 4:**
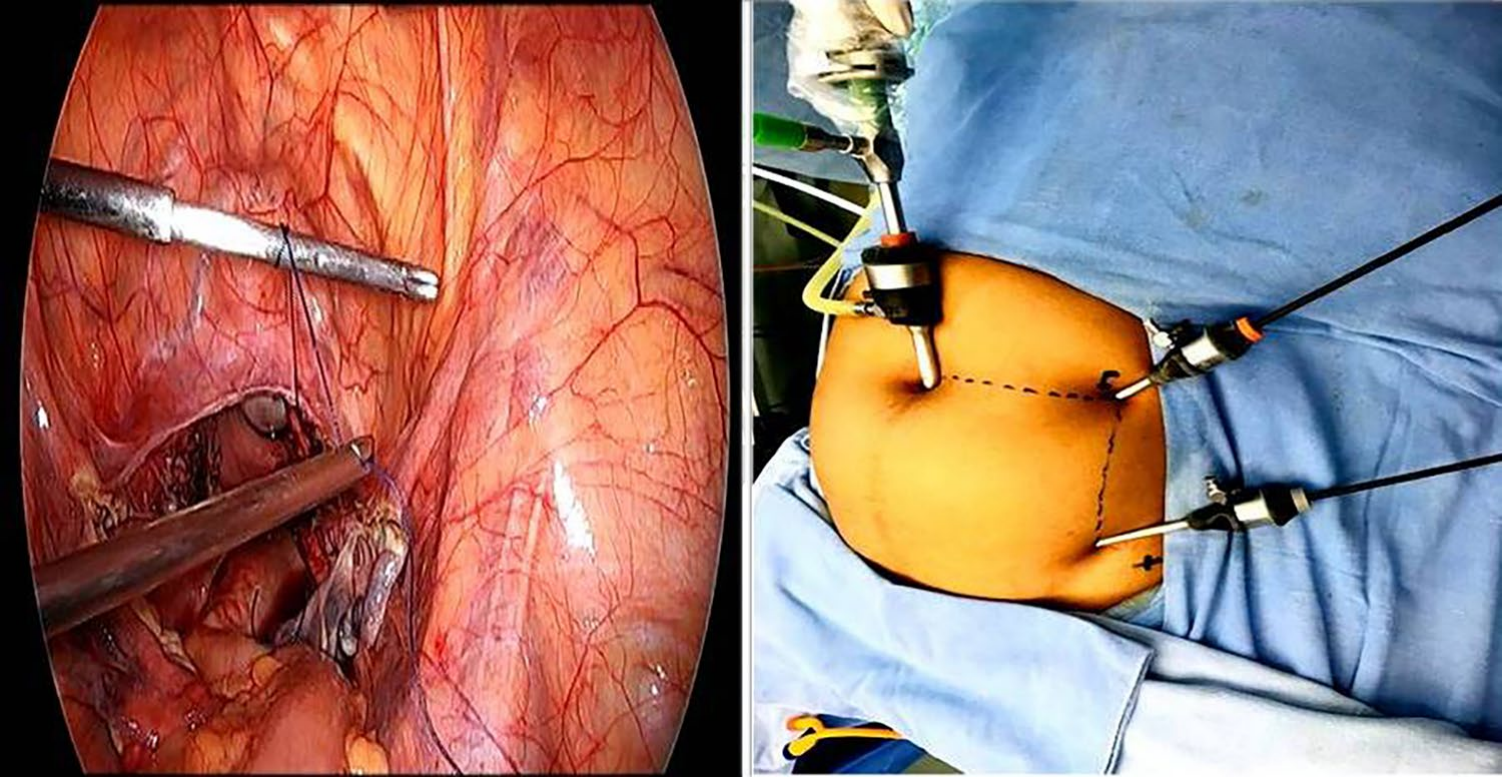
Jain Point port continues as the main ergonomic working port in due course of the surgery

Jain point has an easily located prominent bony land mark in the sterile surgical field, i.e. the anterior superior iliac spine (ASIS). Surface marking of Jain point is that we first locate the ASIS then draw a (Fig. 1) vertical line 2.5 cm medial to ASIS, up to the level of umbilicus, then draw a horizontal line at the upper margin of umbilicus. And, where these two lines meet, that is the Jain point. This point lies at the level of 4th lumbar vertebrae roughly 10–13 cm lateral to the umbilicus depending on the patient’s BMI and body types. The patient is laid in supine position, keeping the plane of the operation table parallel to the floor. A small nick of 2 mm is made just enough for Veress needle entry. Skin at Jain point is made taught between index and middle finger of left hand. Without lifting the abdominal wall, the Veress needle is inserted perpendicular to the abdominal wall, in a vertical direction irrespective of patient’s BMI. We put a finger guard on the Veress needle according to the anticipated thickness of the abdominal wall. As the needle is passed, two distinct pops are heard. The first pop is heard, when the Veress needle passes through the external oblique aponeurosis. The second pop is heard when it pierces the fused aponeurosis of internal oblique and transverse abdominis muscle followed by resistance-free passage of needle in peritoneal cavity. The routine safety check of drop test and low initial Veress intra-peritoneal pressure test (LIVIP) [8] is done before starting CO_2_ insufflation. If high insufflation pressure is noticed, abdominal wall may be lifted to disengage the Veress needle tip from the omentum. With insufflation pressure of 25 mm Hg and about three litres gas flown in the abdomen, a 5 mm trocar and cannula with open vent is inserted with screwing movement, through the Jain point. A zero degree 5 mm telescope is put into the abdomen through the 5 mm trocar. Thorough inspection of whole abdomen and pelvis is carried out and a 10 mm telescope is introduced under visual guidance (Fig. 2), at umbilicus or in upper abdomen according to the need of the case. In our technique, avoiding umbilicus as first blind entry point gives us safety against injury to VVAB (vessel, viscera, adhesions and bowel). (Fig. 3) The Jain point port is then used as the main ergonomic working port throughout the course of the surgery as also mentioned in the article by HT Sharp [1]. (Fig. 4) Being situated at the level of umbilicus, it can also be used in situations where Palmer’s Point is contraindicated as in upper abdominal previous surgery scars, bloated stomach, large upper abdomen gastropancreatic masses, hepatosplenomegaly and portal hypertension.

### Data collection and statistical analysis

The data were collected from Vardhman Trauma & Laparoscopy Centre Pvt. Ltd hospital’s operation theatre records. To study the comprehensiveness of Jain point, patient’s demographic profile like age, BMI, parity, number of previous surgeries, weight of solid masses, and type of laparoscopic surgeries performed and complications encountered during or after primary port insertion were recorded. The data analysis was done using the Statistical Package for Social Sciences (SPSS version 21). Continuous variables such as age and body mass index (BMI) were presented as mean ± 2 SD, whereas quantitative variables like parity and previous surgery status was presented as frequency, relative frequency and range, type of surgeries performed and entry related complications were presented as frequency and relative frequency.

## Results

A total of 7802 laparoscopic surgeries were included in this study. The demographic data are summarised in Appendix Table 1. Types of surgeries performed are shown in Table 2. Weight of the solid masses removed is given in separate Table.3.

**Table 1:** Demographic profile of patients who underwent laparoscopic surgeries. (January 2011 to December 2020)

Characteristics	Values	Median	Range
Age (years)	32.79 + 15.2^a^	31	8–76
BMI (kg/m^2^)	25.30 + 9.52^a^	24.90	12.66–54.41
< 18.5	419 (5.37)^b^		
18.5–24.99	3610 (46.27)^b^		
25–29.99	2694 (34.52)^b^		
30–39.99	994 (12.72)^b^		
> 40	85 (1.08)^b^		
Parity			
Nullipara	4085 (52.67)^b^	–	–
Multipara	3717 (47.32)^b^	2	1–4
Previous abdominal surgeries			
No previous surgery	5561 (71.3)^b^	–	
Previous one surgery	1744 (22.5)^b^	–	
Previous two surgeries	371 (4.6)^b^	–	–
≥ 3 previous surgeries	126(1.5) ^b^	3	3–6

*BMI* body mass index

^a^Mean + 2 standard deviation

^b^Absolute number (percentage)

**Table 2:** Types of surgery performed

S. N	Types of surgery performed (January 2011 to December 2020)	Total number- 7802	With prev. surgery- 2241
1	TLH	963	346
2	Myomectomy	899	199
3	Adenomyomectomy	96	35
4	Endometriosis		
	Gr. III/IV endometriosis/DIE	967	265
	Gr. I/II endometriosis	444	141
	Scar endometriosis	14	14
	Bladder endometriosis	6	3
5	Ovarian cystectomy		
	Ovarian cyst	304	51
	Dermoid cyst	97	24
	Post TLH ovarian cyst	12	12
6	Pelvic floor repair	146	43
7	Mullerian anomalies	146	43
8	Ectopic	333	121
10	Koch’s	1145	451
11	Tubal block	533	200
12	Recanalization	27	20
13	Vaginoplasty	15	0
14	Burch colposuspension	27	0
15	Para vaginal repair	10	2
16	Presacral neurectomy	15	1
17	Lap for pelvic pain	97	58
18	2nd look procedures	121	121
19	Diagnostic laparoscopy infertility evaluations	1372	86
20	Appendectomy	6	2
21	Cholecystectomy	2	1
22	Other	5	2

We are giving details of the procedures carried out and have given a separate table for weight of myomas and uteri

**Table 3:** Cases with solid masses (TLH & myomectomy)

Cases with solid masses (TLH & myomectomy)			Total
Weight of specimen (in g)	TLH	Myomectomy	
Less than 300 g	669	488	1157
300– < 500 g	126	188	314
500– < 1000 g	112	147	259
1000– < 1500 g	33	37	70
1500– < 2000 g	8	19	27
≥ 2000 g	13	20	33
Total	961	899	1860

**Fig. 5:**
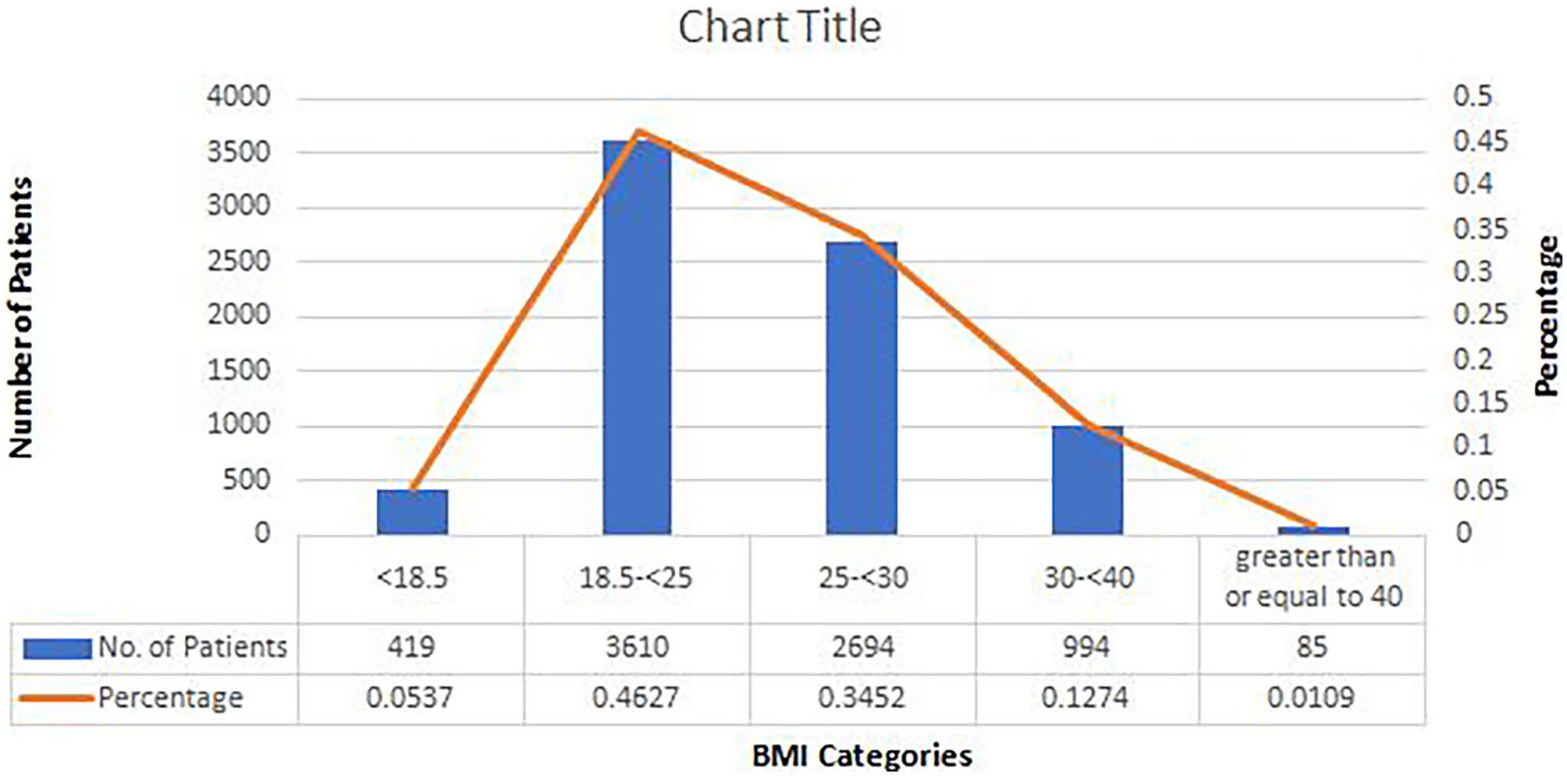
BMI profile of Patients

On average, the age of the patients was around 33 years. The BMI ranged from 12.66 kg/m^2^ to 54.41 kg/m^2^. Only half of the cases (46.27%) belonged to normal BMI range whereas one-third of cases (34.52%) were overweight. A prominent number of patients were obese (12.72%) and morbidly obese (1.08%), and the size of undernourished patients (5.37%) was also noticeable. (Fig. 5) As per parity statistics, around 4085 women were nulliparous, and 3717 women were multiparous. Based on previous abdominal surgeries records, it is evident that in 71.27% women, no previous surgery was done. However, almost one-third of the cases (28.72%) were with previous surgery, either laparoscopy or laparotomy.

The endoscopic surgeries performed at our institute ranged from as simple as infertility evaluation to as complex as deep infiltrating endometriosis, pelvic floor repair and large pelvic masses. It is noteworthy that no case was converted into open surgery, and no mortality was reported, in this study period.

Minor complications noticed were like subcutaneous and omental emphysema due to occasional undershoot and overshoot of veress needle which is quickly detected by higher intra-abdominal pressure in first 10 s (LIVIP) [8] and occasionally failed entry that too in the beginning of learning curve, which settles within few procedures.

There was no major vessels injury. One bowel injury was seen in a case of advanced Koch’s, where small bowel injury was noticed in a case of Koch’s abdomen with previous history of open surgery by a transverse scar at the level of umbilicus. We preoperatively had a very high index of suspicion of adhesions and got an MRI done which showed no bowel beneath the scar, and then we took her for laparoscopy. Needless to say, it was not a good reporting as the first 5 mm trocar went straight into the adherent loop of bowel in the frozen pelvis. The incision at the trocar site was enlarged to 3 cm and the bowel injury repaired. The patient was nil orally till bowel sounds returned and patient was discharged on day four post-operatively without any sequelae.

## Discussion

This study presents a novel, non-umbilical, first blind 5 mm entry port through Jain point in large number of different types of laparoscopic surgeries in a routine manner with the aim of avoiding catastrophic complications related to entry. We improvised the first blind entry by introducing a new entry point which is situated 10–13 cm away from the umbilicus, thus minimizing chances of injury to the great vessels. Laparoscopic entry was performed in 7802 surgeries without any significant complications. The mean BMI in our study was 25.30 kg/m^2^. Individuals with extreme body habitus have variable umbilical–aortic bifurcation relationship, thus impose technical challenges in blind entry through the umbilicus [9, 10]. In our study, 4192 (53.72%) cases were operated with abnormal BMI i.e. either low BMI (< 18.5 kg/m^2^) or high BMI (> 25 kg/m^2^). With supine horizontal operation table, the long veress needle can be inserted through the Jain point in obese patients, perpendicular to the horizontal plane safely. There is no need to change the direction of needle during insertion. In thin patients due to very good muscle tone, the entry pops were very easily identified and entry was found to be easy. It is very simple way to avoid umbilicus and major retroperitoneal vessels which could be just 1.5 cm away in thin patients. We published our experience in thin patients [11]. For the same reason, it is useful in situations of lax abdominal wall. The technique of not lifting the abdominal wall, avoids the jagged tracks and undue pressure application over the abdomen, which may cause sudden overshooting of the veress needle.

Previous surgeries often lead to potential risk of damage to the bowel, omentum and viscera through umbilical site entry due to unforeseen adhesions [12]. We operated 2241 cases with history of previous one to a maximum of previous six surgeries and avoided encountering midline adhesions. The anatomical location of Jain point makes it free of adhesions [13–15]. If we look at the peritoneal location of viscera, the kidney and spleen come maximum upto T10–L1 level, whereas at Jain point, we are at L4 level. Lower down the sigmoid colon adheres to the pelvic brim, leaving a large nascent area free of adhesions on the left side which is used to make the Jain point entry at para-umbilical position. We have already published our experience of Jain point entry in 624 patients with previous abdominal surgeries and found this area on left side to be free of adhesions with no significant entry-related complications. It is applicable in low Pfannenstiel incision, midline vertical or paramedian incision. Jain point is especially a boon for upper abdomen scars [14] where various other non-umbilical entry points like Palmer’s point [16, 17], left ninth intercostal space point [18] and Lee Huang point [19–21], are contraindicated. Their use is also contraindicated in hepatosplenomegaly, portal hypertension, history of previous upper abdomen surgery and in patients with full stomach due to faulty nasogastric tube placement [22]. Here, Jain point scores over all other entry ports, as it lies in mid abdomen and can be used in all contraindications of upper abdomen entry. In abdominogenital Koch’s, lot of bowel and other dense adhesions exist between liver and diaphragm in upper abdomen making upper quadrant entry risky by the Palmers or Lee Huang ports [23–31]. In our series there were 1145 cases of Koch’s abdomen where the primary entry is very challenging. Jain point entry can be made in presence of umbilical hernia and previous mesh hernia repairs. We had operated 11 patients with previous history of mesh hernia repair, in whom the size of the mesh was obtained from the records of previous surgery and a safe abdominal entry was done using Jain point. If we compare all existing entry points, Hasson’s open technique has been undoubtedly the best amongst the available techniques till date [32]. However, a recent update on laparoscopic entries in up-to-date opinioned that Hasson may not be the safest [33, 34]. It importantly cannot avoid the type II bowel adhesions where bowel loops are densely stuck to the anterior abdominal wall [35].

In patients where we anticipated dense adhesions on the left side of the abdomen as in previous colostomy scars, drains and colon pull through, we used mirror image of Jain point from the right side. Clearly Palmers point cannot be used from right side of abdomen due to heightened risk of liver injury. This application of Jain point from right side has been used by general surgeons and urologists [36]. Palmers and Lee Huang points being higher in position cannot be used as a working port especially in pelvic surgeries. Jain point being lower in position at L4 level can be safely used in all the above situations and becomes an ergonomic working port in due course of the surgery. Mulayam et al. have reported direct trocar entry from the Jain point [37]. Mohapatra and Bhusan [38] have also reported the benefit of the left lateral port as the main working port as well as the entry port, indicating dual benefit with good ergonomics. In a recent article “Clinical Perspective Concerning Abdominal Entry Techniques” published in JMIG (Journal of Minimally Invasive Surgery), Goodman L, et al. have also illustrated our Jain point as one of the abdominal entry ports [39].

The major complication rate seen in our study was restricted to one bowel injury in a case of complex advanced genital Koch’s, with previous laparotomy which involved injury to the small intestines. There was no major vessel injury. The incidence in our series is lower than that seen with other entry points. A Dutch study involving 51,559 laparoscopic surgeries by closed-entry technique found an average entry related complication rate of 0.044% and 0.031% for visceral and vascular lesions, respectively [40]. We found no case of major retroperitoneal vessel injury or hematoma. Ours being a training institute, with active fellowship programs, the veress needle insertion is carried out by trainees, fellows and consultants. During transition from the technique of umbilical entry, to which trainees are used to, some prepertioneal and omental insufflation and occasional failed entries are seen in initial cases. We have realised that there is a short learning curve of few procedures. Once the rationale and methodology of the technique is well understood, there is a steep fall in such incidences. Every trainee and fellow has welcomed the routine use of non-umbilical first blind entry as it allays anxieties related to first blind umbilical entry port which has potential of sudden catastrophic vessel or bowel injury and they are carrying forward this technique further in their careers.

There is also experience of Jain point usage among other specialists of laparoscopic surgeries. Our unit is a primarily gynaecological unit but we have cases where gall bladder and hernia repairs are done by general surgeons along with TLH or other gynae surgeries. They have used Jain point in a likewise manner. We have demonstrated Jain point in several live surgery workshops and conferences done for the associations like SELSI (Society for Endoscopic and Laparoscopic Surgeons of India) and AMASI (Association of Minimal Access Surgeons of India) which have membership of gynaecologists, urologists, general surgeons and bariatric surgeons. We proposed Jain point as an alternative port when the first three options (umbilicus, Palmer’s point and Lee-Huang point) are not viable. Moreover, Jain point port which is in mid abdomen can be used later on in surgery as the main working port. This feature is in stark contrast to Palmers point, which becomes redundant after initial entry [1] after our demonstration during live surgeries and lectures this paraumbilical port has been accepted, advocated and widely used by several general surgeons as well as urologists in their clinical practice. They have used it in cases wherever they deemed fit in previously scarred abdomen. This a limitation that, at this time, there are no literature reports of Jain point usage by general surgeons.

Also, our study is not without limitations. Though we have a large case series and study period of 10 years but it is retrospective in nature. It needs more multi-centre randomized control trials to give more data about its safe usage.

## Conclusion

Our large series of 7802 patients entered by Jain point as first blind entry proposes that Jain point is a novel, safe 5 mm non-umbilical alternate entry point for laparoscopic first blind entry. It has a readily available fixed bony landmark, the ASIS, in the sterile surgical field making the surface marking easy and accurate. It is versatile and can be used in patients of different ranges of BMI, from simple to complex cases, large masses and cases with previous surgeries. After making initial first blind entry it continues to be used as a main ergonomic working port. We are routinely making non-umbilical entry by Jain point, a new concept aimed at reducing entry related injuries to major retro peritoneal vessels, viscera, adhesions and bowel (VVAB) which could lie under the umbilicus.
